# Risk factors for canine cognitive dysfunction syndrome in Slovakia

**DOI:** 10.1186/s13028-016-0196-5

**Published:** 2016-02-29

**Authors:** Stanislav Katina, Jana Farbakova, Aladar Madari, Michal Novak, Norbert Zilka

**Affiliations:** 1 Department of Mathematics and Statistics, Masaryk University, Kotlarska 2, Brno, Czech Republic; 2 University of Veterinary Medicine and Pharmacy, Komenskeho 73, Kosice, Slovak Republic; 3 Institute of Neuroimmunology, Slovak Academy of Sciences, Dubravska cesta 9, Bratislava, Slovak Republic; 4 Institute of Neuroimmunology, No., Dvorakovo Nabrezie 45, Bratislava, Slovak Republic

**Keywords:** Canine cognitive dysfunction syndrome, Cognitive decline, Risk factors, Prevalence, Epidemiology, Nutrition

## Abstract

**Background:**

Increasing prevalence of cognitive impairment in an aging canine population poses a serious health problem. Identifying risk factors, which may influence the onset of cognitive decline, is becoming increasingly important. Here we investigated whether age, sex, weight, nutrition, dogs’ housing and reproductive state were associated with increased risk of canine cognitive dysfunction syndrome (CCDS) in Slovakia.

**Results:**

Age was associated with cognitive decline and nutrition emerged as a significant predictor variable. Dogs fed controlled diets had 2.8 times lower odds of developing CCDS when compared with dogs fed uncontrolled diets. Sex, weight, reproductive state and dogs’ housing were not significantly associated with cognitive decline. Further, the prevalence of CCDS was similar in both small and medium/large sized dogs aged 8–11 years, but differed in dogs at an age of 11–13 years.

**Conclusion:**

Age was found to be the most prominent risk factors of CCDS. Nutrition may influence the cognitive state of dogs. This finding suggests that nutritional interventions may modify canine cognitive functions.

## Background

Canine cognitive dysfunction syndrome (CCDS) is a complex of behavioural symptoms in aged dogs characterised by deficits in learning, memory, perception, awareness and by impairment of social interactions and sleeping patterns [1]. Improvements in nutrition, elimination of most infectious diseases, better hygiene and adoption of antibiotics and vaccines have increased the life expectancy of dogs. The immediate consequence of the extended life span is an increased number of aged dogs. It has been estimated that there are more than 45 million dogs around 7-years-old in USA and Europe [2]. Because ageing is the main risk factor of CCDS, aged dogs likely represent a large population with a great risk of developing CCDS [3]. Indeed, Neilson et al. [4] showed that the prevalence of CCDS was 28 % in 11–12 years old dogs and 68 % in 15–16 years old dogs. Similarly, Azkona et al. [5] demonstrated that 22.5 % of dogs older than 9 years displayed cognitive impairment.

As a result of the increasing number of cognitively impaired dogs, increasing emphasis is being placed on management and therapy of an age-related canine cognitive impairment [3]. Thus identifying modifiable risk factors and developing preventive strategies for CCDS represent important goals. Currently, there are only limited data available about the risk factors of CCDS, as only a few studies dealing with the risk factors of CCDS have been conducted over the last decade. A study carried out in Spain suggested an association between sex, size, reproductive state and cognitive function of aged dogs [5]. That study showed that females and neutered dogs were significantly more affected than males and intact dogs. Moreover, small breeds had greater odds of showing age-related cognitive impairment than medium or large sized dogs, although weight was not a statistically significant predictor variable. The major drawback of the study lied in structured phone interviews that did not take into account the subjective evaluation of pet owners. Similarly, Hart et al. [6] demonstrated that sexually intact male dogs were significantly less likely than neutered dogs to progress from mild impairment to severe impairment. Another study, conducted in Denmark, showed that neither sex nor weight correlated with cognitive decline [7]. Contradictory results are likely caused by different methodologies used for detection of canine cognitive decline.

The aim of the present study was to test several variables, age, sex, reproductive status, bodyweight, dogs’ housing and nutrition in order to identify possible risk factors for CCDS.

## Methods

### Animals

Study animals were identified among all dogs visiting the Small Animal Veterinary Clinic of the University of Veterinary Medicine and Pharmacy in Kosice, Slovak Republic for regular vaccination, parasite treatments and various health complaints. The age of study dogs ranged from 8 to 18 years (mean = 11 years), there were 116 males and 99 females of different breeds, body weights ranged from 2.3 to 55 kg (mean = 19.6 kg) and reproduction state was neutered males (n = 88), neutered females (n = 79); intact males (n = 25), and intact females (n = 23).

### Haematology and biochemistry

An integral part of the diagnosis was haematological and biochemical blood tests. The following haematological variables were analysed by IDEXX ProCyte Dx^®^ Hematology Analyzer: HCT, RBC, HGB, MCV, MCH, MCHC, red cell distribution width (RDW), reticulocytes (absolute number and percentage), WBC, neutrophils, lymphocytes, monocytes, eosinophils, and basophils (number and percentage), platelets [number, MPV, platelet distribution width (PDW) and PCT], band neutrophils (when presence suspected) and nRBCs (nucleated red blood cells, when presence suspected). Biochemical blood parameters were analysed by Cobas C 111 analyzer (Roche). These variables included ALT, AST, ALP, pAMS, LIP, Crea, UREA, Glu, Chol, TP, Alb, Ca, P, Mg, NH3, K, Na, Cl. All dogs included in this study were assessed by neurological examination, orthopaedic, radiology, ultrasound and ECG examination, as well as by blood and urine analyses. Eighty-five dogs were excluded from the study because of other medical causes interfering with cognitive decline such as blindness, deafness, diabetes mellitus, cushing syndrome, urinary tract infection, incontinence of urine or faeces, cardiological patients, head trauma and other disease conditions.

### Cognitive evaluation

Behavioural investigation included observation of geriatric dogs by a veterinary clinician and collection of information provided by pet owners. The investigator was pro-active in asking about behavioural abnormalities to identify even subtle signs that often go unrecognized by pet owners. Data collected by using questionnaire was important for calculation of the final score. The questionnaire also included basic information about dog characteristics and lifestyle variables such as sex, age, weight, reproductive state, dog’s housing and type of diet. The composite scale–CAnine DEmentia Scale (CADES) used in this study was adapted and modified from the questionnaires proposed by Osella, et al. [8] and Salvin et al. [9]. It contained 17 items distributed into four domains (spatial orientation, social interactions, sleep-awake cycles and house soiling) related to changes in dog’s behaviour. The value of each item corresponded to the frequency of abnormal behaviour. We used a 5-point scale for easy evaluation of behaviour: 0—abnormal behaviour of the dog had never observed; 2—abnormal behaviour of the dog was detected at least once within the last 6 months; 3—abnormal behaviour appeared at least once per month; 4—abnormal behaviour was seen several times per month; 5—abnormal behaviour was observed several times a week. The score from each domain was added up to obtain a final quantitative score that reliably reflected the qualitative evaluation of cognitive decline. We validated CADES as a screening tool for CCDS [10]. Dogs were divided into two subgroups: dogs with no or very mild cognitive impairment (MiCI) and dogs with an advanced cognitive decline. The first group consists of cognitively normal dogs (NA; CADES score 0–7) and dogs with MiCI (CADES score 8–23). The second group consists of dogs with moderate cognitive impairment (MoCI; CADES score 23–44) and dogs with severe cognitive impairment (CADES score higher than 44).

### Prevalence calculation

The prevalence of CCDS in three age groups was calculated. All tested dogs were divided into age groups based on estimated life expectancies [11]: short-lived, 6–11 years; medium-lived, 11–13 years and long-lived, >13 years, as proposed by Salvin et al. [9]. The prevalence was calculated by dividing the number of dogs in each tested group (NA, MiCI, MoCI, CD) by the number of individuals examined in each age category.

### Data analysis

Statistical analyses were performed with R software [12]. Univariable logistic regression analysis (two-sample *Z*-test of log odds) [13] was used to assess relationships between cognitive impairment as dependent variable (moderate and severe CCDS vs normal ageing and mild cognitive impairment) and risk factors such as sex (males vs females), reproductive state (neutered vs entire dogs), food (uncontrolled-scrabs, commercial dry or wet food low quality or mix of different kind of food vs controlled diet—commercial dry or wet food for specific breed, age or life stages—obese, neutered, intact, working dogs), dog’s housing (outside—dogs spending much of their time outside the house vs inside—dogs spending much of their time inside the house or flat) and weight (under 15 kg vs over 15 kg, under 15 kg means smaller or equal than 15 kg in the paper) as independent variables. All age and weight related intervals used in the text are open from left and closed from right, e.g. 11–13 years means greater than 11 and smaller or equal than 13 (except for the first in the range, which is closed from both sides, e.g. 8–11 years means greater or equal than 8 and smaller or equal than 11). The risk was considered to be positive with respect to cognitive impairment when the estimate of odds ratio (OR) was greater than one, and negative when OR was less than one. Additionally, Wald 95 % empirical confidence intervals for OR were calculated. To test correlation between age (in years) and composite CADES score, one-sample Fisher *Z*-test of zero correlation was used. Additionally, Wald 95 % empirical confidence intervals for Pearson product-moment correlation coefficient were calculated. All of the null hypotheses were tested against two-sided alternatives on significance level α = 0.05. Finally we also used saturated additive multivariable logistic regression model with interaction (of sex and reproductive state) of the form-cognitive impairment ~ sex + reproductive state + diet + housing + weight + (sex: reproductive state), where the log odds were tested similarly as in univariate analyses equivalent to univariable logistic regression model.

## Results

### Correlation of age and CCDS

**Fig. 1:**
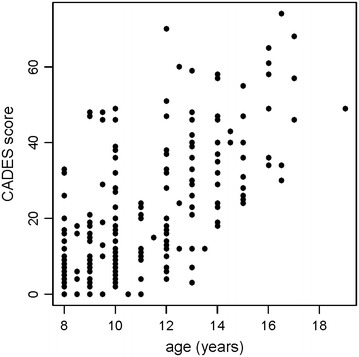
The positive correlation of CADES score and age of dogs

First, we focused on relationship between advancing age and CADES score reflecting degree of cognitive impairment. There was positive correlation between both advancing age and CADES score (estimate of Pearson product-moment correlation coefficient *r* = 0.662, *t* = 12.895, *df* = 213, *P* < 0.0001, 95 % CI: (0.580, 0.731); Fig. 1).

### Association between nutrition and CCDS

For the study of putative risk factors, the dog population with a slightly uneven sex ratio [54 % males, 46 % females] was classified as follows: weights—small size breeds (less than or equal to 15 kg; 112 dogs) or medium/large size breeds (over 15 kg; 103 dogs), nutrition-controlled diets (113 dogs) or uncontrolled diets (102 dogs), dogs’ housing-outside (78 dogs) or inside (137 dogs) and reproductive status—neutered (167 dogs) or entire dogs (48 dogs); see Table 1.

**Table 1:** Distribution of dogs throughout selected categories and stages of canine dementia

Categories	Total number of dogs	Weight		Nutrition		Sex		Dogs‘ housing		Reproductive status	
		Over 15 kg	Below 15 kg	Uncontrolled diet	Controlled diet	Females	Males	Inside	Outside	Neutered	Entire
Normal ageing	56	31	25	21	35	30	26	34	22	43	13
Mild cognitive impairment	80	38	42	31	49	37	43	52	28	64	16
Moderate cognitive impairment	49	23	26	30	19	19	30	32	17	38	11
Severe cognitive impairment	30	11	19	20	10	13	17	19	11	22	8

**Fig. 2:**
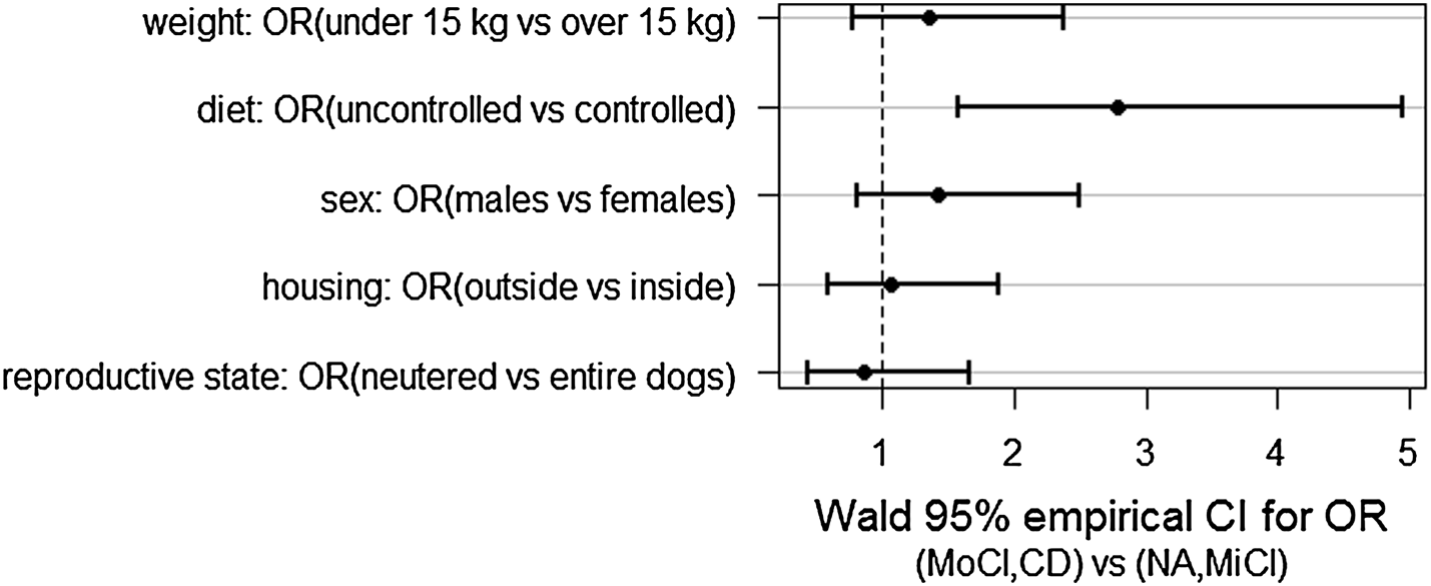
Nutrition represents the only risk factor for CCDS identified in the study

Univariable analyses demonstrated that there was a significant association between nutrition and CCDS (*P* < 0.001). Dogs fed with controlled diet displayed 2.8 times lower odds of CDS when compared to dogs fed uncontrolled diets (OR = 2.8). Sex (*P* = 0.11), weight (*P* = 0.14), reproductive status (*P* = 0.32) and dogs’ housing (*P* = 0.42) were not significant at the univariable level (Table 2; Fig. 2). Multivariable analyses showed similar results. Sex, reproductive state, housing, weight, and interaction of sex and reproductive state were all ruled out from saturated model and the only statistically important variable is diet (OR = 2.8, *P* < 0.001).

**Table 2:** The results of statistical analyses of the risk factors—weight, nutrition, sex, dogs’ housing and reproductive state for canine dementia

Variable (group 1 vs group 2)	n_1_	N_1_	n_2_	N_2_	OR	sd (OR)	LB	UB	*Z*-statistics	*P* value
Weight OR (under 15 kg vs over 15 kg)	45	112	34	103	1.36	0.21	0.78	2.38	1.0879	0.1383
Nutrition OR (uncontrolled vs controlled diet)	50	102	29	113	2.79	0.82	1.57	4.94	3.5006	*0.0002*
Sex OR (males vs females)	47	116	32	99	1.43	0.20	0.81	2.50	1.2401	0.1075
Dogs’ housing OR (outside vs inside)	51	137	28	78	1.06	0.28	0.59	1.89	0.1943	0.4230
Reproductive state OR (neutered vs entire dogs)	60	167	19	48	0.86	0.39	0.44	1.65	−0.4627	0.3218

*n*_*1*_ the number of moderate and severe cognitive impaired dogs in group 1 (weight below 15 kg, nutrition-uncontrolled diet, males, housing inside, neutered), *n*_*2*_ the number of moderate and severe cognitive impaired dogs in group 2 (weight above 15 kg, nutrition-controlled diet, females, housing outside, entire), *N*_*1*_, *N*_*2*_ total number of dogs in group 1 and group 2, *OR* odds ratio, *sd* (*OR*) standard deviation of odds ratio, *LB* lower bound, *UB* upper bound of Wald 95 % empirical confidence interval (CI) for OR, *Z-statistics* two-sample *Z*-test about log OR (*P* values related to statistically significant results are highlighted in italics; the bounds of CI are back transformed bounds of CI for log OR)

### The prevalence of CCDS increased with age

**Fig. 3:**
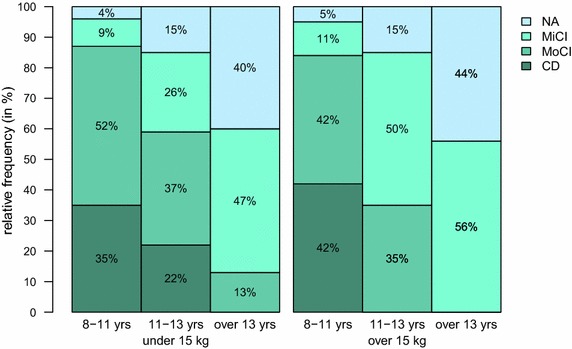
The prevalence of CCDS. *Barplots* demonstrate that the prevalence of CCDS increases with age. *NA* normal ageing, *MiCI* mild cognitive impairment, *MoCI* moderate cognitive impairment, *CD* severe cognitive impairment

We found that the prevalence of fully developed CCDS (MoCI + CD) was 13 % (7 dogs, below 15 kg) and 16 % (12 dogs, over 15 kg) in the age period 8–11 years, 41 % (11 dogs, below 15 kg) and 65 % (13 dogs, over 15 kg) in the age period 11–13 years and 87 % (26 dogs, below 15 kg) and 100 % (9 dogs, over 15 kg) in the age over 13 years. Further, 52 % (28 dogs, below 15 kg) and 42 % (31 dogs, over 15 kg) of 8–11 years old dogs and 37 % (10 dogs, below 15 kg) and 35 % (7 dogs, below 15 kg) of 11–13 years old dogs were classified as mildly affected (MiCI). Prevalence of CCDS was similar in both small and medium/large sized dogs in the age period 8–11 years (13 vs 16 %), and differed in older dogs, 11–13 years old (41 vs 65 %); see Table 3; Fig. 3. In dogs older than 13 years, we did not find any cognitively intact dog.

**Table 3:** The prevalence of CCDS

Current paper			
	8–11 years	11–13 years	Over 13 years
Small breed (%)	13	41	87
Medium/large breed (%)	16	55	100
Salvin et al. [9]			
8–10 years	10–12 years	12–14 years	Over 14 years
3.4 %	5 %	23.3 %	41 %
Azkona et al. [5]			
	9–11 years	12–14 years	15–17 years
Small breed (%)	22.7	32.7	50.0
Medium/large breed (%)	10.3	26.9	40

## Discussion

The cognitive decline of aged dogs represents serious medical and social problem. Increasing number of dogs older than 7 years is leading to a higher prevalence of CCDS worldwide [2]. The aging profile of dogs varies between breeds, generally smaller dogs can live up to 10–14 years, while medium and large sized breeds live up to 8 years [14]. Today, dogs are generally living longer than previously thanks to increasing knowledge about nutritional needs and advances in veterinary medicine. Increasing life span does not equal increasing life quality as shown by higher prevalence of age-related disorders or healthy problems such as arthritis and joint problems, prostate enlargement, urinary incontinence, constipation, hearing and vision loss.

Several studies have shown that the prevalence of CCDS raises with increased age [4, 5, 8, 9]. However, telephone interviews used in some studies cannot exclude various medical causes of CCDS and thus the prevalence of CCDS can be overestimated. To strengthen the design of the study, we combined structured interviews with pet owners with direct clinical evaluation. Previously, Salvin et al. [9] showed that the prevalence of CCDS increased with age. Similarly, Azkona et al. [5] demonstrated increased prevalence of CCDS in both small breeds and medium/large breeds. In contrast, we observed greater prevalence of CCDS in medium/large sized breeds compared with small sized breeds in the age group of 11–13 years, while no difference was observed in the age group of 8–11 years. Our findings are in accordance with a previous study that focused on the relationship between body size and life span [14]. The researchers clearly demonstrated that while there was no clear correlation between body size and the onset of senescence, there was a strong positive relationship between size and aging rate. Finally, they concluded that dogs of large breeds died younger than small breed dogs, mainly because they aged more quickly. We plan to increase the total number of dogs to identify whether some of the breeds are more vulnerable to CCDS than the others.

The increasing incidence of CCDS highlights the importance of identification of putative aetiological risk factors. Unfortunately, knowledge regarding risk factors of CCDS is still quite limited and some data are rather contradictory, e.g., one study found that sex and size of breeds had no association with CCDS [7], while another study indicated that small sized dogs and females had a greater risk of developing signs of CCDS [5]. Another proposed risk factor was the reproductive state. Azkona et al. [5] demonstrated a higher risk of CCDS in neutered female and male dogs in comparison to intact dogs. Similarly, Hart [6] showed that sexually intact male dogs were significantly less likely than neutered dogs to progress from mild impairment to severe impairment. Surprisingly, our data did not support the contribution of sex, reproductive status and weight to the disease progression. We also did not find any correlation between dogs’ housing and cognitive decline. In case of dogs’ housing and reproduction state, we are aware of the fact, that the number of dogs in each group (neutered vs entire, outside vs inside) is not equal. In our study, we recruited much more neutered dogs than intact dogs and the majority of dogs lived inside of houses or flats. This uneven distribution may have influenced the outcome of the study.

On the other hand, we found for the first time that nutrition may represent an important factor for development of CCDS. We defined two groups of diets as proposed by Hand et al. [15]: controlled diet and uncontrolled food. In this study, controlled diet is characterized as high-quality commercial food purchased for the size of the breed or age or healthy status demands (Hill’s, Royal Canine, Specific etc.). Uncontrolled diet represents kitchen waste, unspecified feed mixture or low-quality commercial food, which is not purchased for the size of the breed or age or healthy status demands. Our study revealed that uncontrolled diets may increase a dog’s risk of developing CCDS. Based on our results we suggest that controlled diets could be more protective against cognitive damage than homemade or mixed diets. Our findings may explain why nutritional manipulation through the use of special diets and dietary supplements may be beneficial for treatment and prevention of CCDS [16]. We suggest that CCDS is an aging-related multi-factorial disorder that may be modified by a properly balanced diet.

We are aware of strengths and weaknesses of our study. While this study included only a small number of subjects and conducted in one clinic in Slovakia, and therefore, may represent regional and/or cultural group, the results are consistent with several independent nutritional studies showed that controlled diet can significantly improve, or slow the decline of learning and memory in aged dogs [17, 18]. Moreover, it has been demonstrated that antioxidants and mitochondrial cofactors, and long-term supplementation with medium-chain triglycerides improve cognitive function in aged dogs [19].

## Conclusions

We demonstrated that the prevalence of CCDS almost tripled in small dogs and quadrupled in medium/large dogs, within the age period from 8 to 13 years. We also confirmed previous studies showing that age represent the main risk factors for CCDS. We found that nutrition may represent important factor in the development of CCDS at least in the dog population of Slovakia. Further studies are warranted to support this finding. This study further highlights the urgent need for effective preventative strategies and therapeutic approaches.

## Abbreviations

Alb: albumin
ALT: alanine aminotransferase
ALP: alkaline phosphatase
AST: aspartate aminotransferase
CADES: canine dementia scale
CCDS: canine cognitive dysfunction syndrome
Chol: cholesterol
Crea: creatine
DISHA: disorientation, interaction changes, sleep/wake disturbances, house soiling and activity changes
ECG: electrocardiography
Glu: glucose
HCT: haematocrit
LIP: lipase
RBC: red blood cells
HGB: haemoglobin
MiCI: mild cognitive impairment
MCI: human mild cognitive impairment
MCH: mean corpuscular hemoglobin
MCHC: mean corpuscular hemoglobin concentration
MCV: mean corpuscular volume
MoCI: moderate cognitive impairment
MPV: mean platelet volume
NA: normal ageing
nRBCs: nucleated red blood cells
OD: odds ratio
PCT: plateletcrit
pAMS: amylase
PDW: platelet distribution width
RDW: red cell distribution width
TP: total protein

## References

[1] Landsberg GM, Nichol J, Araujo JA (2012). Cognitive dysfunction syndrome: a disease of canine and feline brain aging. Vet Clin North Am Small Anim Pract.

[2] Bosch MN, Pugliese M, Gimeno-Bayón J, Rodríguez MJ, Mahy N (2012). Dogs with cognitive dysfunction syndrome: a natural model of Alzheimer’s disease. Curr Alzheimer Res.

[3] Sanabria CO, Olea F, Rojas M. Cognitive dysfunction syndrome in senior dogs. In: Kishore U, editor. Neurodegenerative diseases, 2013. p. 615–628.

[4] Neilson JC, Hart BL, Cliff KD, Ruehl WW (2001). Prevalence of behavioral changes associated with age-related cognitive impairment in dogs. J Am Vet Med Assoc.

[5] Azkona G, García-Belenguer S, Chacón G, Rosado B, León M, Palacio J (2009). Prevalence and risk factors of behavioural changes associated with age-related cognitive impairment in geriatric dogs. J Small Anim Pract.

[6] Hart BL (2001). Effect of gonadectomy on subsequent development of age-related cognitive impairment in dogs. J Am Vet Med Assoc.

[7] Fast R, Schütt T, Toft N, Møller A, Berendt M (2013). An observational study with long-term follow-up of canine cognitive dysfunction: clinical characteristics, survival, and risk factors. J Vet Int Med.

[8] Osella MC, Re G, Odore R, Girardi C, Badino P, Barbero R, Bergamasco L (2007). Canine cognitive dysfunction syndrome: prevalence, clinical signs and treatment with a neuroprotective nutraceutical. Appl Anim Beh Sci.

[9] Salvin HE, McGreevy PD, Sachdev PS, Valenzuela MJ (2010). Under diagnosis of canine cognitive dysfunction: a cross-sectional survey of older companion dogs. Vet J.

[10] Madari A, Farbakova J, Katina S, Smolek T, Novak P, Weissova T, Novak M, Zilka N (2015). Assessment of severity and progression of canine cognitive dysfunction syndrome using CAnine DEmentia Scale (CADES). Appl Anim Behav Sci.

[11] Michell AR (1999). Longevity of British breeds of dog and its relationships with sex, size, cardiovascular variables and disease. Vet Rec.

[12] R Development Core Team (2014). R: A language and environment for statistical computing.

[13] Lachin JM. Biostatistical methods. The assessment of relative risks. Hoboken: Wiley & Sons; 2011.

[14] Kraus C, Pavard S, Promislow DE (2013). The size-life span trade-off decomposed: why large dogs die young. Amer Nat.

[15] Hand MS, Thatcher CD, Remillard RL, Roudebush P (2010). Small animal clinical nutrition.

[16] Zicker SC (2005). Cognitive and behavioral assessment in dogs and pet food market applications. Prog Neuropsychopharmacol Biol Psychiatry.

[17] Head E, Rofina J, Zicker S (2008). Oxidative stress, aging, and central nervous system disease in the canine model of human brain aging. Vet Clin North Am Small Anim Pract.

[18] Pan Y (2011). Enhancing brain functions in senior dogs: a new nutritional approach. Top Companion Anim Med.

[19] Manteca X (2011). Nutrition and behavior in senior dogs. Top Companion Anim Med.

